# Evidence of putative persistence of bacterial spores in a dairy processing environment

**DOI:** 10.3168/jdsc.2026-1133

**Published:** 2026-10-05

**Authors:** Wendy Bollum, Nicole Martin

**Affiliations:** Department of Food Science, Cornell University, Ithaca, NY 14853

## Abstract

Bacterial spores, which are found in raw milk and are able to survive high temperature, short time pasteurization, are a well-documented concern for dairy product spoilage. Due to their resistance and propensity to occupy environmental niches, bacterial spores may also be found in food processing environments, ultimately leading to introduction into processed dairy products through cross-contamination, although few studies have investigated the presence of dairy-relevant spores in processing environments. Here, we aim to enumerate and identify predominant bacterial spores isolated from 14 environmental sites in a dairy processing facility over 3 independent sampling events. To that end, mesophilic spore tests (i.e., mesophilic spore count and mesophilic most probable number spore test) were performed on the environmental sponge samples (n = 42). Sponges collected from the processing facility were heat treated at 80°C for 12 min, followed by both a most probable number technique to enumerate low-level contamination, as well as pour plating on standard methods agar to evaluate a broad range of potential spore levels. The mean spore count across the 14 environmental sites ranged from −1.34 log most probable number/mL to 3.86 log cfu/mL with SD of 1.47 log cfu/mL, demonstrating large differences across sites in both concentration and variability. General purpose tables and a microthermics benchtop had the highest mean spore counts, whereas the exterior of the spray dryer powder nozzle and the exterior of the HTST pasteurizer showed the lowest mean spore counts. Isolates representing unique colony morphologies were characterized using *rpoB* gene sequencing. The 133 isolates successfully sequenced comprised 14 genera, 42 species, and 55 unique *rpoB* allelic types, including 53 isolates that represented 25 new allelic types not previously described. The most common genera isolated were *Bacillus* and *Paenibacillus*, representing 34.6% and 22.6% of the isolates, respectively. Other genera included *Cytobacillus* (15.8%), *Niallia* (8.3%), and others (18.8%). Of the *Bacillus* isolated from the processing environment, *Bacillussubtilis s.l. Clade1* and *Bacillus cereus* group were the most commonly isolated. Further, we observed sporeformers with the same *rpoB* allelic types over multiple sampling events from the same sites, suggesting putative persistence in the processing environment. Overall, our results indicate that dairy processing environments may harbor spores of relevance to dairy product quality and safety. Future studies should further examine spores in processing environments and develop strategies to prevent their introduction into dairy products to reduce food waste, extend product shelf life, and reduce risk to public health.

## Graphical Abstract

**Figure:**
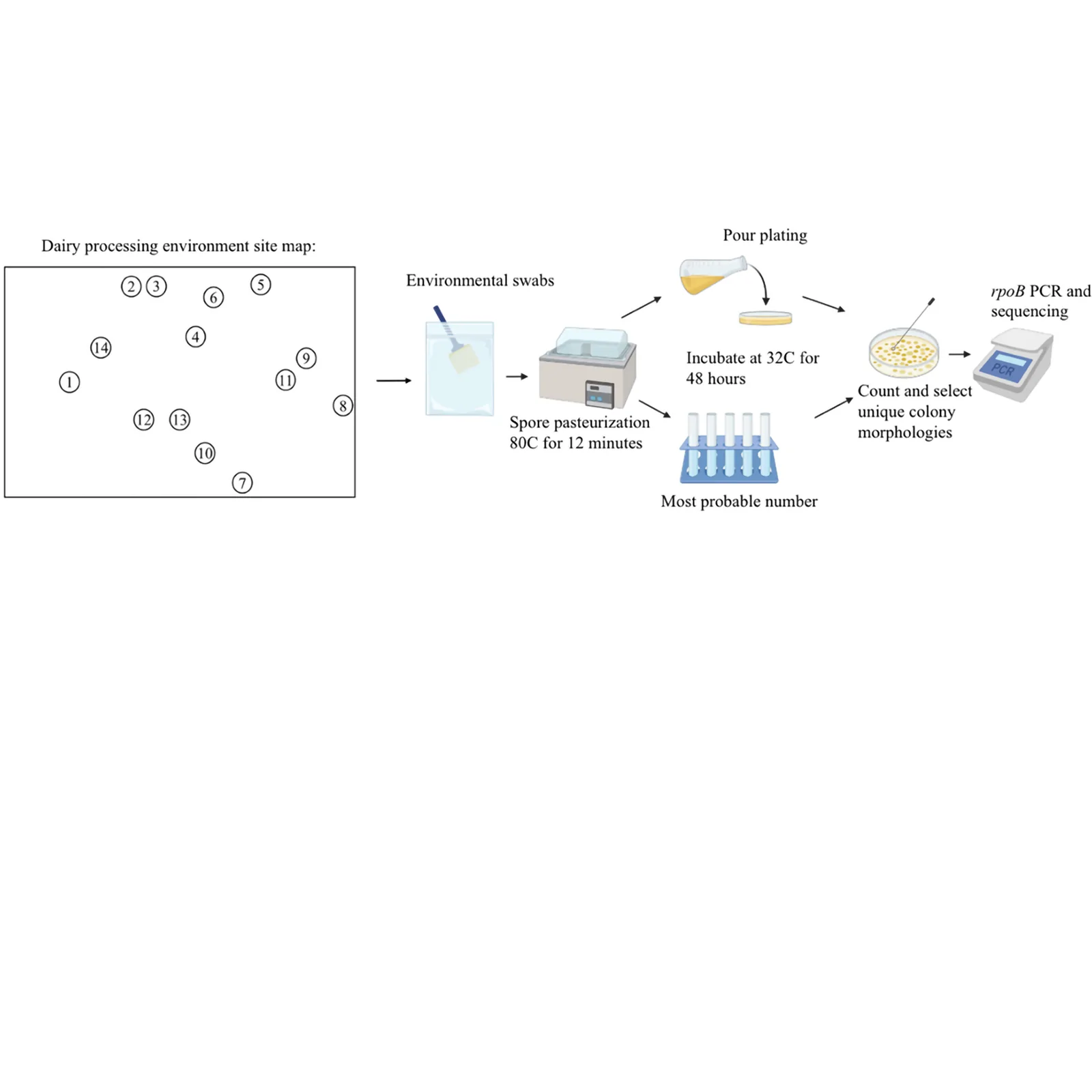

Sporeforming bacteria negatively affect the quality and shelf life of dairy products and may also cause foodborne illness. Premature microbial spoilage and subsequent food loss is a relevant concern to producers, processors, and consumers. Spores are ubiquitous in natural environments and can be introduced during production, where spores enter milk due to inadequate udder hygiene and related milking practices (Evanowski et al., 2020). Bacterial spores may also be introduced at the processor level through insufficient equipment cleanliness and sanitation, or cross-contamination with biofilms (Martin et al., 2021). Common genera of spoilage-causing sporeforming bacteria include *Bacillus* and *Paenibacillus* in fluid milk and *Bacillus* and *Geobacillus* in dairy powders (Ranieri and Boor, 2009; Miller et al., 2015). The most relevant pathogenic sporeformer in dairy products is *B. cereus* *s.s.*, although a recent outbreak of *Clostridium botulinum* in infant formula (Chung et al., 2026) highlights the need for a renewed focus on other pathogenic sporeforming bacteria in the dairy system as well.

Given their ubiquitous nature, their resistance to common control measures used in the dairy industry (e.g., pasteurization), and the very low level of spores needed to cause negative impacts on finished products, the introduction of bacterial spores into products needs to be studied at every stage in the dairy continuum. Although spores have been extensively studied at the farm level and in some manufacturing equipment (Doll et al., 2017; Evanowski et al., 2023), there is far less literature outlining their presence in the manufacturing environment, with only one such study that characterized isolates (n = 467) from processed foods, raw materials, and dairy processing environments (Lücking et al., 2013). Due to the importance of the manufacturing environment for other dairy-relevant bacterial contaminants (e.g., *Listeria*, *Cronobacter*, and so on), it should not be overlooked as a reservoir of sporeforming bacteria relevant to spoilage and human health. Therefore, the aim of this study was to enumerate and identify heat-resistant bacterial spores isolated from 14 environmental sites in a dairy processing facility over 3 sampling trials.

The environmental sites (n = 14) in a pilot plant facility that processes both dairy and nondairy products were selected to represent a wide range of potential niches for bacterial spores (Table 1). There were 3 sampling events for the 14 sites, each conducted approximately 1 wk apart from one another. Environmental swabs (Neogen, Lansing, MI) were used to swab an approximately 10-inch by 10-inch (25.4-cm by 25.4-cm) square at each site. For sites where it was difficult to swab a continuous area of this size (e.g., exterior of a spray dryer powder nozzle), the largest area representing the distinct site was swabbed. Swabs were immediately transported to the Milk Quality Improvement Program laboratory (Ithaca, NY), where 90 mL of brain heart infusion (**BHI**; Becton, Dickinson and Company, Franklin Lakes, NJ) broth was added to each sponge bag. The bags were stomached at 270 rpm for 1 min. Mesophilic spore tests were performed on the environmental samples (n = 42) as described in the following text. Samples were heat treated by holding the sponge bag in a shaking water bath at 80°C for 12 min. This heat treatment served to eliminate vegetative bacteria and activate germination of spores. The samples were subsequently cooled in an ice bath until they reached 6°C, and then 1 mL of the sample broth was pour plated in duplicate using BHI agar. In addition, to enumerate low-level spore contamination (i.e., samples with plates with no colonies), a most probable number (**MPN**) technique was used in addition to the direct count described in the preceding text. Briefly, 10, 1, and 0.1 mL of the heat-treated sample broth were added to 0, 9, and 9 mL, respectively, in triplicate to achieve a 3-tube dilution scheme of each volume of BHI broth. Both the pour-plated mesophilic spore counts (**MSC**) and the MPN tubes were incubated at 32°C for 24 h before enumeration or, in the case of MPN tubes, scoring for growth. The MPN outcome was only used for samples that were below the detection limit with the direct plate count method. Because swabbed surface area varied among sites and MPN estimates were used when direct counts were below detection, results are presented descriptively as log concentrations in the recovery buffer and are intended to characterize relative variation within this facility. For averages and SD of concentrations from different samplings or different sites, if both cfu/mL and MPN/mL units were present, the unit representing the greater number of samples was retained.

**Table 1:** Mesophilic spore counts (log cfu/mL or MPN/mL) from swab samples collected in a processing plant environment

Site no.	Site location	Sampling A	Sampling B	Sampling C	Mean	SD
1	General purpose table 1	3.13	2.63	2.67	2.81	0.22
2	Equipment cover 1	1.54	1.32	1.57	1.48	0.11
3	Equipment cover 2	1.85	1.50	1.02	1.45	0.34
4	General purpose table 2	3.42	4.21	3.96	3.86	0.33
5	Microthermics benchtop	3.64	3.17	3.76	3.52	0.25
6	Stainless steel kettle	0.70	0.56	−0.28	0.33	0.43
7	General purpose sink	2.15	2.06	2.10	2.10	0.03
8	Stainless steel storage tank adjacent to processing room	1.78	2.02	1.12	1.64	0.38
9	Exterior of spray dryer powder nozzle	−2.52^1^	0.72	−1.64^1^	−1.15^2^	1.37
10	General purpose table 3	1.30	0.00	0.72	0.67	0.53
11	Moveable storage cart	−1.64^1^	2.06	1.60	0.68	1.65
12	Exterior of HTST pasteurizer	−2.52^1^	−2.52^1^	1.02	−1.34^2^	1.67
13	Exterior of raw milk holding vat	0.70	2.06	2.10	1.62	0.65
14	Computer keyboard	2.45	2.45	2.35	2.42	0.05

1 Samples with no detectable colonies on direct plate count have been substituted with MPN estimation.

2 Means that contain 2 MPN/mL values and 1 cfu/mL value are represented as MPN/mL.

Following incubation, samples with positive results on the direct count method and positive MPN tubes were further subjected to bacterial isolation. From MPN tubes, positive tubes were substreaked onto BHI agar and incubated for 24 h at 32°C. From these plates as well as any positive direct count plates, isolates representing unique colony morphologies were selected and streaked for purity on BHI agar and incubated at 32°C for 24 h. Selecting colonies with unique morphologies for further characterization is an approach that is commonly used to identify representative isolates in a given sample (Huck et al., 2007; Ivy et al., 2012); however, it should be acknowledged that this approach may result in overrepresentation or underrepresentation of certain strains depending on colony morphology characteristics.

Molecular subtyping of all isolates was performed based on a 632-nucleotide fragment of the *rpoB* gene, with primers previously described (Drancourt et al., 2004). The *rpoB* gene has been shown to have improved discriminatory power over the 16S rRNA gene and has been used extensively for species level assignment and subtyping of aerobic sporeforming bacteria (Gaballa et al., 2021). For isolates that failed initial *rpoB* gene subtyping (i.e., did not amplify or were not successfully sequenced; n = 25), a ∼700-nucleotide fragment of the 16S rRNA gene was amplified with forward primer 16S-PEU7 and reverse primer 16S-DG74 (Lee et al., 2024). Sanger sequencing was performed at Cornell University's Biotechnology Resource Center (Ithaca, NY). The generated forward and reverse sequences were trimmed, aligned, and edited using Sequencher (Gene Codes Corporation, Ann Arbor, MI). All sequences were assigned a genus, and *rpoB* sequences were also assigned species and allelic type (**AT**), based on the existing Food Microbe Tracker database (Gaballa et al., 2021). A complete list of bacterial isolates collected from this study, along with metadata and sequencing data, is cataloged at https://www.foodmicrobetracker.net/, and is searchable by the title of this publication.

Overall, we observed high levels of variability across the 14 environmental sites tested here. The mean spore count across the 14 environmental sites ranged from −1.34 log MPN/mL to 3.86 log cfu/mL and the site means had an overall SD of 1.47 log cfu/mL (Table 1). The sites with the highest spore concentrations were general purpose table 2, microthermics benchtop, and general purpose table 1, with means of 3.86, 3.52, and 2.81 log cfu/mL, respectively (Table 1). The sites with the lowest mean spore concentrations were the exterior of the HTST pasteurizer, the exterior of the spray dryer powder nozzle, and the stainless steel kettle, with mean spore counts of −1.34 log MPN/mL, −1.15 log MPN/mL, and 0.33 log cfu/mL, respectively (Table 1).

In addition to between site variation, our results indicate that bacterial spores in the food manufacturing environment vary considerably within the same location over time in some cases. The sites with the highest variability in spore count across sampling events were the exterior of the HTST pasteurizer, the moveable storage cart, and the exterior of the spray dryer powder nozzle, with SD of 1.67, 1.65, and 1.37 log cfu/mL, respectively (Table 1). Conversely, the sites with the lowest SD across sampling events were the general purpose sink, the computer keyboard, and equipment cover 1, with SD of 0.03, 0.05, and 0.11 log cfu/mL (Table 1). Although this is the first study to our knowledge describing bacterial spore levels in a processing environment, variability of other dairy-relevant microorganisms has been studied extensively in processing environments. For example, yeasts and molds are well-known contaminants in dairy products originating from the processing environment. One study collected 102 aerosol samples over a 12-mo period from across a dairy processing facility (Beletsiotis et al., 2011). Yeast and mold counts between the sampling sites ranged from 93.6 to 266.2 cfu/m^3^. They proposed that this variability is due to differing humidity, temperature, dust load, and proximity to the exterior door of the sample sites, as well as species adaptation to the indoor microenvironment. Wilson et al. took environmental samples in duplicate from 25 locations in a washed-rind cheese processing plant and found variable fungal counts as high as 5.65 log cfu/swab on the wooden planks in the aging room and 5.19 log cfu/swab from a floor drain, whereas many of their other samples from tables and lines were below their limit of quantification of 3.0 log cfu/mL (Wilson et al., 2024). The variable nature of the fungal counts for the drain swabs is likely due to their biweekly (every 2 wk) cleaning schedule, where they saw much lower values after cleaning. As for the planks in the aging room, higher fungal counts were associated with more frequent rind washing and longer duration since the rind washing cart had been cleaned. Overall, variability in microbial concentration in processing facilities is likely driven by the interval that cleaning and sanitation are performed, with some sampling site characteristics such as moisture, temperature, and proximity to exterior doors also playing a role. Future studies should further explore the variability of bacterial spore concentrations in dairy processing environments to identify intrinsic characteristics of the environment (e.g., moisture, residual sanitizer concentrations, and so on) that may be associated with locations exhibiting high spore concentrations, or high variability in spore concentration.

In total, from all 42 samples, 148 bacterial isolates were collected, 133 of which were successfully characterized (n = 123 using *rpoB* gene sequencing and n = 10 using 16S rDNA sequencing), and 15 that were not successfully characterized. The 133 successfully characterized isolates included in taxonomic analyses represented 14 genera, 42 species, and 55 *rpoB* AT, including 53 isolates that represented 25 new AT not previously described (Table 2). The remaining 15 isolates that could not be characterized were excluded from genus-, species-, and allelic-type summaries. The most common genera were *Bacillus* and *Paenibacillus*, representing 34.6% and 22.6% of the isolates, respectively. Other genera included *Cytobacillus* (15.8%), *Niallia* (8.3%), and others (18.8%).

**Table 2:** Characterization of sporeforming bacterial isolates (n = 133) collected from environmental samples in a processing facility (n = 123 for *rpoB* allelic typing and n = 10 for 16S rRNA sequencing)

Organism	*rpoB* AT	No. of isolates
*Bacillus*		46
*amyloliquefaciens*	1,710	1
*bataviensis*	N/A^1^	1
*cereus* group	120	1
*cereus* group	2,583^2^	5
*cereus* group	N/A^1^	1
*cereus s.s.*	92	5
*licheniformis*	1	4
*licheniformis*	6	1
*paranthracis*	125	2
*safensis*	N/A^1^	1
sp. 4	271	1
sp. 4	282	2
*subtilis s.l. Clade1*	211	6
*subtilis s.l. Clade1*	330	10
*subtilis s.l. Clade1*	583	1
*subtilis_murimartini s.l.*	66	1
*velezensis*	360	3
*Brevibacillus*		4
*parabrevis*	1,131	1
sp.	N/A^1^	3
*Cytobacillus*		21
*eiseniae*	591	1
*eiseniae*	974	3
*eiseniae*	2,591^2^	4
*eiseniae*	2,592^2^	1
*eiseniae*	2,607^2^	1
*firmus*	2,599^2^	1
*kochii*	207	1
sp.	2,589^2^	9
*Fictibacillus*		1
sp.	2,602^2^	1
*Lysinibacillus*		9
*capsici*	2,595^2^	1
*fusiformis*	71	3
*pakistanensis*	2,584^2^	1
*pakistanensis*	2,598^2^	1
sp. 4	367	1
sp. 4	2,605^2^	1
sp. 7	2,586^2^	1
*Metasolibacillus*		1
*meyeri*	1,206	1
*Niallia*		11
*alba*	460	3
*alba*	510	1
*taxi*	2,582^2^	2
*taxi*	2,590^2^	5
*Oceanobacillus*		1
*caeni*	392	1
*Paenibacillus*		30
*camelliae*	2,585^2^	1
*campinasensis*	379	1
*dakarensis*	2,608^2^	1
*glucanolyticus*	159	13
*humicus*	1,929	4
*pseudotheri*	1,945	1
sp.	2,593^2^	1
sp.	2,597^2^	1
sp.	2,601^2^	1
sp.	N/A^1^	3
sp. 4	2,596^2^	1
*typhae*	845	1
*xylanilyticus*	1,394	1
*Priestia*		1
*megaterium*	2,466	1
*Robertmurraya*		1
*korlensis*	64	1
*Solibacillus*		3
sp.	2,581^2^	1
sp.	2,606^2^	2
*Sporosarcina*		3
*newyorkensis*	2,594^2^	2
sp.	2,588^2^	1
*Terribacillus*		1
*saccharophilus*	2,587^2^	1

1 N/A = not available; isolates where the internal region of the 16S rRNA gene was sequenced, not *rpoB*.

2 Isolates representing *rpoB* AT newly identified in this study.

Of the *Bacillus* isolated from the processing environment, *B. subtilis s.l. Clade1* was the most commonly isolated, followed by *B. cereus* group. The presence of *Bacillus cereus* group is particularly important, as it is a species complex with members that represent both quality and food safety concerns to the dairy industry. For example, *B. cereus sensu stricto* is the causative agent of foodborne illness from the production of toxins either in the food itself or in the digestive tract after being consumed (Su et al., 2024). One source estimates the number of illnesses due to *B. cereus* is 63,400 annually in the United States, but the actual number is likely underreported due to the short-lived nature and mild symptoms of the illness (Scallan et al., 2011). As for spoilage, *Bacillus weihenstephanensis*, a member of the *B. cereus* group, is responsible for a defect in fluid milk known as sweet curdling, where proteolytic activity from the bacteria causes coagulation (Martin et al., 2021). Other members of the family also produce thermo-resistant enzymes that result in product spoilage (e.g., off-flavor, rancidity, bitterness; Tirloni et al., 2022). Of relevance to our study, *B. cereus* is shown to produce biofilms on surfaces common in the processing environment (e.g., stainless steel, plastic, glass), with a carbon source and NaCl (Kwon et al., 2017). Growth in these conditions suggests *B. cereus* may grow in the food processing environment, increasing the risk of contaminating product. Eneroth et al. (2001) attributed the contamination of pasteurized fluid milk to *B. cereus* contamination of a filling machine, whereas another study attributed the source of *B. cereus* contamination to pasteurization equipment (Eneroth et al., 2001; Svensson et al., 2000). Given the relevance of *B. cereus* to dairy product quality and safety, its presence in processing equipment demonstrated by previous studies and in the processing environment demonstrated in our study suggests that further investigation of potential transmission pathways from environments into finished dairy products should be a priority.

The second most frequent genus isolated was *Paenibacillus*, of which *P. glucanolyticus* was the most prevalent with 13 isolates (Table 2). *Paenibacillus glucanolyticus* is a predominant psychrotolerant sporeformer that causes spoilage in dairy products via its production of proteases, lipases, and phospholipases. These enzymes result in product defects, including off-flavors, bitterness, and curdling (Grady et al., 2016). *Paenibacillus*, along with *Bacillus*, accounts for 40% to 50% of fluid milk that reaches the Pasteurized Milk Ordinance (**PMO**) limit of 20,000 cfu/mL during refrigerated storage (Martin et al., 2021). The PMO is a set of sanitary standards in the United States for grade A milk, one of which is that pasteurized milk must not exceed 20,000 cfu/mL. In addition, one study characterized isolates from pasteurized milk over shelf life and found that 92% of the gram-positive sporeforming isolates characterized at d 17 were *Paenibacillus* (Ranieri and Boor, 2009). Similar to our results, in the one existing study that characterized sporeforming bacteria from the processing environment, the authors note that 75% of the isolates from the processing environment (n = 103) and product (n = 266) were *Bacillus* and 6% were *Paenibacillus* (Lücking et al., 2013). The authors also report *B. cereus s.l.* as the species group most frequently encountered, representing 23% of isolates, followed by *B. licheniformis*, *B. subtilis*, and *Geobacillus stearothermophilus*, at 19%, 10%, and 8% of isolates, respectively (Lücking et al., 2013). These studies highlight the relevance of *Paenibacillus* as a prominent spoilage organism in dairy products and, with its presence in the dairy processing environment found in our study, potential risk for product contamination should be considered.

Notably, 53 of the sporeformer isolates in this study represent new AT not previously characterized. This may indicate that the processing environment represents a unique niche where sporeforming bacteria adapt to pressures (e.g., sanitizers, low moisture, and so on) that result in taxonomic distinction. Further characterization of these isolates should be conducted to understand if the presence of advantageous stress response genes related to survival in processing environments is overrepresented in these isolates.

Finally, we observed isolates with the same *rpoB* AT over multiple sampling events from the same sites, indicating putative persistence in the processing environment. Three *rpoB* AT, AT2591 (*Cytobacillus eiseniae*), AT2589 (*Cytobacillus* sp.), and AT2590 (*Niallia taxi*), were each isolated from a single site over all 3 sampling events (Figure 1). One *rpoB* AT, *Paenibacillus glucanolyticus* (AT159), was isolated 2 times across 3 sites each and one time across 4 sites each (Figure 1). While this repeated recovery of the same subtypes across multiple sampling events suggests the possibility of putative persistence, we cannot rule out reintroduction of genetically similar subtypes into the environment across time.

**Figure 1:**
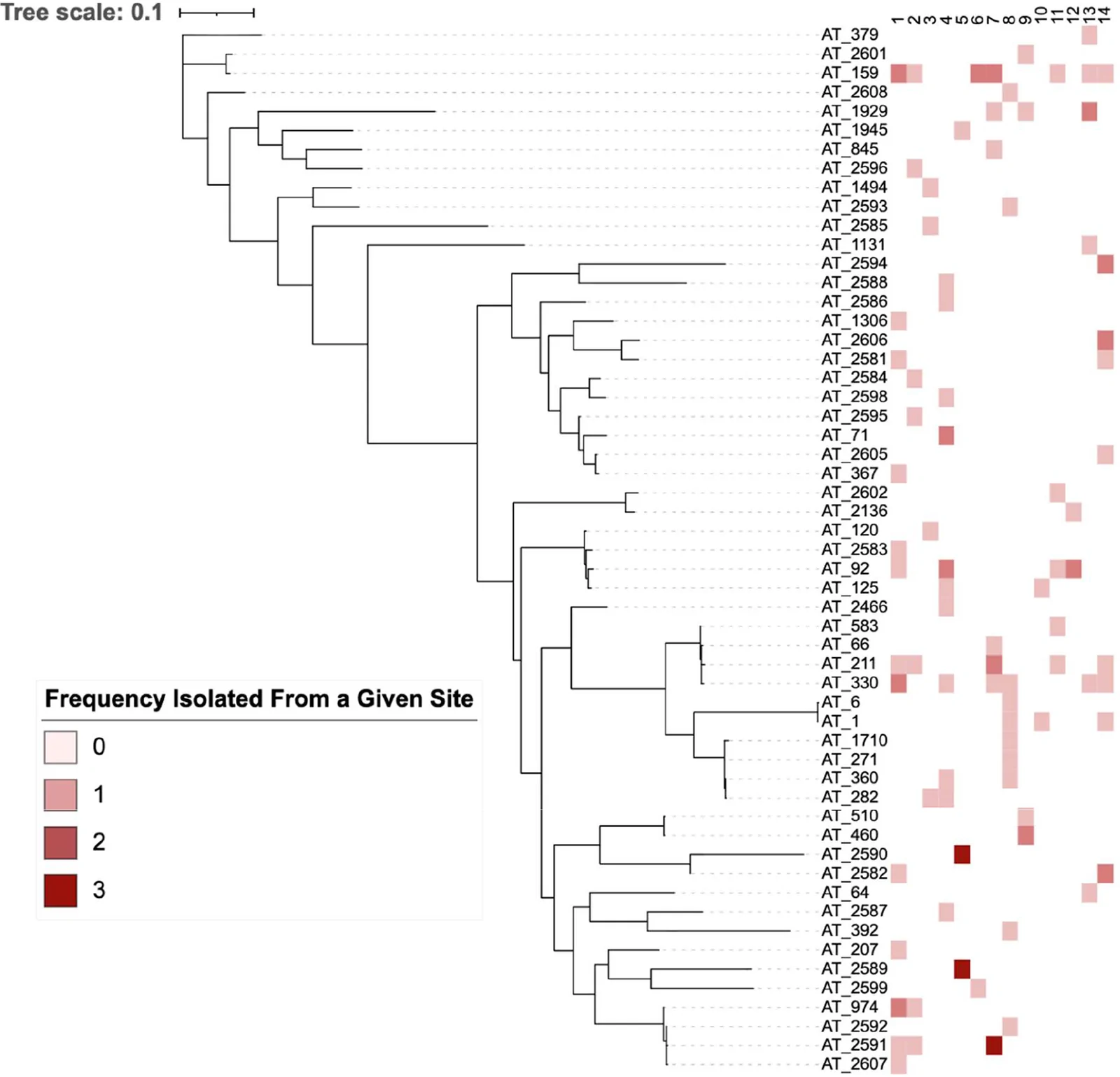
Phylogenetic tree of all sporeforming bacterial isolates successfully sequenced using the *rpoB* gene by allelic type (AT; n = 123), shown vertically, with a heat map of frequency of isolation by sampling site, shown horizontally. The sampling sites, 1 to 14, are listed across the top of the figure and correspond to those outlined in Table 1. Frequency of isolation is indicated in white for an allelic type never isolated from that site, increasing in color intensity by number of times isolated to dark red for the same allelic type isolated across all 3 samplings. Repeated recovery of the same allelic type isolated from all 3 samplings is interpreted as putative persistence in the text.

Repeated discovery of the same AT raises the possibility that protected environmental niches, cleaning and sanitation practices, or repeated introduction may contribute to their continued detection. Although no previous research has evaluated persistence of sporeforming bacteria in dairy processing environments, persistence of other microorganisms in dairy processing environments has been widely studied, in particular, the environmental pathogen *Listeria monocytogenes*. This pathogen survives in many niches in the food processing environment, even those with low humidity, temperature, or oxygen content (Bucur et al., 2018). One study of biofilm formation by *L. monocytogenes* found that temperature was the most influential factor in biofilm formation, compared with salt content and nutrient availability, positively correlated as the temperature nears 37°C (Lee et al., 2019). Related, when looking at the strength of adhesion of *L. monocytogenes* biofilms, cells that were exposed to nutrient deprivation or cold stress showed strong initial adhesion of the biofilm (Lee et al., 2019). Biofilm formation is an important factor for persistence of vegetative bacteria, and there is robust research, especially for *L. monocytogenes*, on what conditions encourage their growth in the processing environment. While no studies specifically evaluate biofilm formation by sporeformers in dairy processing environments, a recent study of 196 sporeforming bacteria found that 29.6% of the strains tested formed strong biofilms, with 18% and 64% of the 2 most commonly isolated species, *B. cereus* and *B. licheniformis*, forming strong biofilms, respectively (Medjahdi et al., 2025). Further research is needed to investigate conditions that lead to the presence of sporeforming bacteria in dairy processing environments and factors that may increase risk of persistence.

In conclusion, bacterial spores in the dairy processing environment represent a potentially important and understudied area of research. Here, we enumerated and characterized sporeforming bacteria in a pilot plant processing environment. We found both high and temporally variable MSC, putative persistence from multiple *rpoB* AT in multiple locations, and *rpoB* AT belonging to taxa previously associated with dairy product quality, safety, and conformance. Given this study was meant to be exploratory and not comprehensive, there are a few limitations to our data. Namely, (1) only 14 sites in one dairy processing facility were sampled, and (2) the schedule of swabbing did not take into account the cleaning and sanitation schedule of the plant, which could affect the presence and levels of spores in the environment. Further research should be conducted to understand the populations, persistence, transmission into dairy products, and intervention strategies for effectively controlling spores in the dairy processing environment.
